# Evaluation of agricultural non-point source pollution using an in-situ and automated photochemical flow analysis system

**DOI:** 10.1038/s41598-024-65251-6

**Published:** 2024-06-23

**Authors:** Yongqi Chen, Muhammad Awais, Junfeng Wu, Zhenfeng Li, Syed Muhammad Zaigham Abbas Naqvi, Mukhtar Iderawumi Abdulraheem, Hao Zhang, Ling Wang, Wei Zhang, Vijaya Raghavan, Jiandong Hu

**Affiliations:** 1 Department of Electrical Engineering, Henan Agricultural University, Zhengzhou, 450002 China; 2 Henan International Joint Laboratory of Laser Technology in Agriculture Science, Zhengzhou, 450002 China; 3 State Key Laboratory of Wheat and Maize Crop Science, Zhengzhou, 450002 China; 4 Department of Bioresource Engineering, Faculty of Agriculture and Environmental Studies, McGill University, Sainte-Anne-de-Bellevue, QC H9X 3V9 Canada

**Keywords:** Agricultural non-point source pollution (ANPS), FDR soil water content sensor, Automatic photochemical flow analysis, Sampling well, Porous ceramic probe, Electrical and electronic engineering, Environmental monitoring

## Abstract

Off-line leachate collection from agricultural landscapes cannot guarantee precise evaluation of agricultural non-point source (ANPS) due to geospatial variations, time, and transportation from the field to the laboratory. Implementing an in-situ nitrogen and phosphorous monitoring system with a robust photochemical flow analysis is imperative for precision agriculture, enabling real-time intervention to minimize non-point source pollution and overcome the limitations posed by conventional analysis in laboratory. A reliable, robust and in-situ approach was proposed to monitor nitrogen and phosphorous for determining ANPS pollution. In this study, a home-made porous ceramic probe and the frequency domain reflectometer (FDR) based water content sensors were strategically placed at different soil depths to facilitate the collection of leachates. These solutions were subsequently analyzed by in-situ photochemical flow analysis monitoring system built across the field to estimate the concentrations of phosphorus and nitrogen. After applying both natural and artificial irrigation to the agricultural landscape, at least 10 mL of soil leachates was consistently collected using the porous ceramic probe within 20 min, regardless of the depth of the soil layers when the volumetric soil water contents are greater than 19%. The experimental results showed that under different weather conditions and irrigation conditions, the soil water content of 50 cm and 90 cm below the soil surface was 19.58% and 26.08%, respectively. The average concentrations of NH_4_^+^-N, NO_3_^−^-N, PO_4_^3−^ are 0.584 mg/L, 15.7 mg/L, 0.844 mg/L, and 0.562 mg/L, 16.828 mg/L and 0.878 mg/L at depths of 50 cm and 90 cm below the soil surface, respectively. Moreover, the comparison with conventional laboratory spectroscopic analysis confirmed R^2^ values of 0.9951, 0.9943, 0.9947 average concentration ranges of NH_4_^+^-N, NO_3_^−^-N, and PO_4_^3−^, showcasing the accuracy and reliability of robust photochemical flow analysis in-situ monitoring system. The suggested monitoring system can be helpful in the assessment of soil nutrition for precision agriculture.

## Introduction

Agricultural non-point source (ANPS) is a prevalent form of pollution that exerts a significant influence on water systems, encompassing rivers, lakes, and groundwater^1–4^. In contrast to pollution originating from concentrated sources such as factories, non-point source pollution originating from dispersed sources across agricultural landscapes presents a greater challenge in terms of identification and localization^5,6^. Chemical fertilizers, insecticides, and herbicides are recognized as prominent agents that contribute to ANPS pollution^7,8^. Livestock has been recognized as an additional contributing factor to the occurrence of ANPS pollution^9,10^. Wastes with high nitrogen (N) and phosphorus (P) contents not only feed aquatic systems but also trigger potentially dangerous algal blooms and environmental disruptions^11,12^.

Globally, it has been well recognized that ANPS pollution is posing hazardous effects on agricultural landscapes and water bodies worldwide. Moreover, an estimated 75% of these affected areas have experienced nitrogen (N) and phosphorus (P) pollution^13^. The average nitrogen utilization has been found to exceed the internationally recommended safe threshold resulting in the occurrence of cluster, explosion, and crossover ANPS pollution^14^. Moreover, it has been observed in several other developed nations that ANPS pollution has emerged as a significant contributor to water pollution^15^. Additionally, ANPS pollution from agricultural lands, such as nutrients, sediment, and agricultural chemicals, can originate from multiple locations, making it difficult to implement centralized control measures^16^.

The majority of nitrogen (N) and phosphorus (P) measurement techniques involve using of photonic-chemical analysis in a laboratory setting after the collection of soil leachates^17–19^. A variety of methodologies have been developed to measure the presence of nitrogen and phosphorus in soil, such as Fourier transform infrared spectroscopy (FTIR), Raman spectroscopy, and near-infrared spectroscopy (NIR)^20^. Although FTIR, Raman spectroscopy and NIR have the advantages of high detection accuracy, wide detection range and short detection time^21,22^. The detection equipment is precise and vulnerable to damage in the complex environment of farmland, which cannot meet the needs of in-situ detection in farmland. Therefore, these measurement methods that are currently accessible necessitate the collection of soil leachates, their subsequent transportation, and their analysis later on in the well-designed laboratory. The assessment of ANPS pollution poses challenges in terms of data collection, measurement techniques, soil leachate transportation, and the spatial variability of N and P causing the majority of the ANPS pollution. Hence, these methodologies exhibit deficiencies in terms of precision, in-situ setting, and resilient assessment of ANPS pollution levels^23^.

This paper proposes a reliable, robust and automatic photochemical flow analysis approach to monitor nitrogen and phosphorous for determining ANPS pollution. In this study, a home-made porous ceramic probe and the FDR water content sensors were strategically placed at different soil depths to facilitate the collection of leachates. These solutions were subsequently analyzed by automatic photochemical flow analysis monitoring system built across the field to estimate the concentrations of phosphorus and nitrogen. Using in-situ sampling and automated detection in conjunction with a sampling well provides numerous benefits in terms of temporal efficiency and mitigating soil leachate variability during the transportation process to the laboratory. This, in turn, enhances the overall efficiency and accuracy of ANPS pollutants’ detection processes^24^. The aforementioned system promptly acquire soil leaching solution in-situ, and it is imperative that the gathered leaching solution not be transported to the well-designed laboratory for the purpose of assessing and analyzing soil leachates. This guarantees that it will not be varied and protects it from any transportation requirements. As a result, this in-situ monitoring system greatly improves testing accuracy and automation while also reducing the difficulties and costs related to transportation.

The automatic photochemical flow analysis and monitoring system can monitor non-point source pollution without affecting the mechanized farming of farmland. The automatic photochemical flow analysis and monitoring system can eliminate the lag and uncertainty of the detection results of traditional detection methods, and understand the impact of ANPS pollution on agricultural ecosystem in the context of climate change more comprehensively and accurately^25,26^.

## Materials and methods

### Materials

The FDR (MT10, frequency domain reflectometer) were acquired from Dalian Zheqin Technology Co. for the purpose of measuring soil water contents. These FDR sensors possess a robust construction and are equipped with a waterproof design, rendering them highly suitable for extended periods of continuous detection in challenging environmental conditions^27^. Subsequently, the soil water content sensors were carefully positioned inside a cylindrical receptacle, which was fabricated using advanced three-dimensional (3D) printing techniques. This meticulous construction approach was employed to guarantee the container’s ability to consistently retain accurate measurements^28^. The homemade porous ceramic probe utilized for the purpose of collecting the soil leaching solution is composed of a diverse assemblage of minerals, encompassing kaolinite, hydromuscovite, montmorillonite, quartz, and feldspar. These exceptional properties encompass, air permeability, water permeability, and a diverse range of other distinctive characteristics^29^. The characteristics mentioned above serve to guarantee the successful separation of the leachate from the soil, thus enabling an optimal leaching process. The coordination of the piping and valve networks was executed in a manner that prioritized the seamless operation of the entire photochemical flow analysis monitoring system. The chemical reagents utilized in this study were obtained from Wuhan Huashen Chemical Reagent Co. Ltd., located in China.

### In-situ ANPS monitoring system

**Figure 1:**
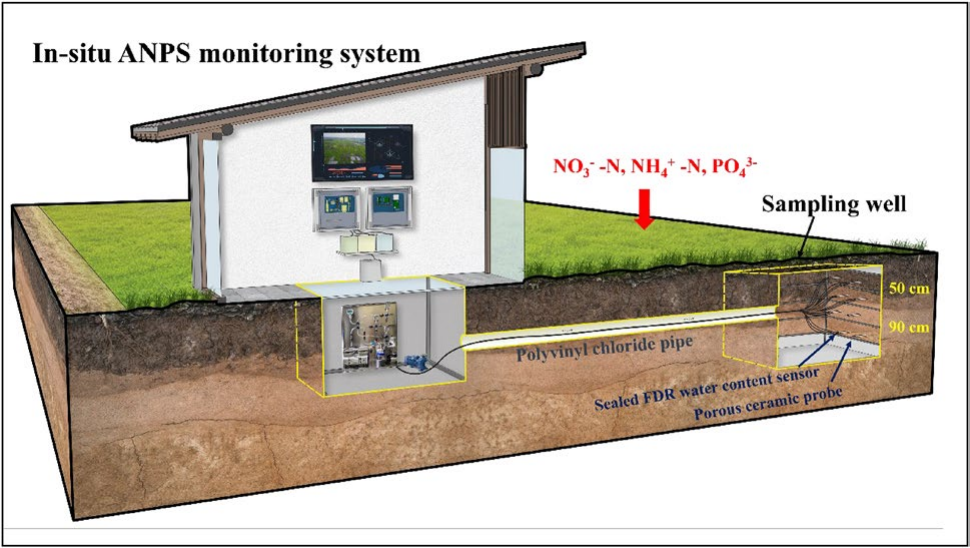
Deployment of automatic collection system of soil leachate and measurement of NH_4_^+^-N, NO_3_^−^-N, PO_4_^3−^ in agricultural landscape to monitor ANPS pollution.

Determining the movement of nitrate nitrogen and phosphorus between different soil depths is crucial for evaluating non-point source pollution because it helps to understand the pollution sources, transport mechanisms, and potential mitigation strategies. The transport of nitrate nitrogen and phosphorus in the soil is influenced by various factors, such as soil texture, water contents, and the irrigation. Nitrate nitrogen and phosphorus can readily leach from the soil into surface and groundwater, leading to non-point source pollution^30^. Generally, the location adapted for the placement of the automatic photochemical flow analysis monitoring system was strategically situated at the intersection of the agricultural landscape. The photochemical flow analysis monitoring system is purposed for in-situ soil leaching solution collection and automated measurement for the assessment of ANPS pollution in agricultural landscapes. Figure 1 depicts the schematic diagram of the automatic photochemical flow analysis monitoring system and also explains the accumulation of ammonium nitrogen (NH_4_^+^-N), nitrate nitrogen (NO_3_^−^-N), phosphorus (PO_4_^3−^) and transportation from various non-point sources. The excessive runoff of N and P disrupts natural nutrient balances, soil acidification, and is toxic to the ecosystem when they become volatile. The sampling well construction is also presented in Fig. 1, which advocates the idea of keeping the soil layers intact, making the soil leachate collection non-invasive.

The system employed for collecting the leaching solution from various soil layers in an agricultural landscape involved the utilization of porous ceramic probes. The in-situ monitoring system for the measurements of NH_4_^+^-N, NO_3_^−^-N, PO_4_^3−^ consisted of several components, including a modular photochemical detection unit, three switching valves, two negative air and positive air pumps, an ultrasonic liquid position sensor, and a temporary bottle for the soil leaching solution.

### Soil leaching solutions collection

**Figure 2:**
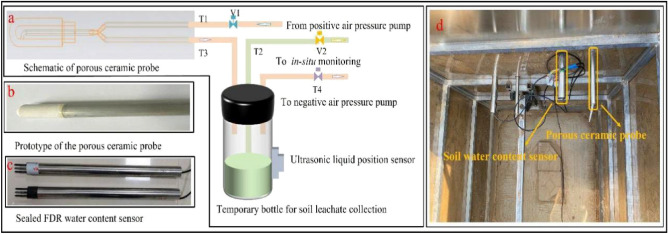
The architecture for soil leachate collection and the sampling well located in Henan Agricultural University farm. (**a**) Schematic diagram of soil leaching solution collection and storage. (**b**) Prototype of the porous ceramic probe, (**c**) the sealed FDR soil water content sensor and (**d**) the photograph of the sampling well depicted the process of drilling a hole in the wall.

This positioning facilitated the effective and streamlined collection of soil leaching solutions from the sampling well. The soil leachates were acquired from the Henan Agricultural University’s farm facility situated at coordinates 34°47′ N, 113°38′ E. The choice of the sampling location was determined by its significant agricultural history, which is marked by the cultivation of many crops like maize, sweet potato, chili, and beans^31^. The farm soil type is sandy, consisting of small particles with a high water saturation. In this study, a sampling well with a dimension of 120 × 120 × 200 cm was excavated to attain an intact soil profile throughout the surrounding area of the sampling well. From the wall of the sampling well, holes were drilled horizontally into the soil layers, exposing the porous ceramic probe opening (Fig. 2a). A galvanized square steel welded frame was used to construct this structure to ease drilling operations and withstand pressure from the surrounding soil. Two porous ceramic probes and one FDR soil water content sensor were arranged in the same soil layer (Fig. 2b, c). After that, the enclosed structure was slowly lowered and fixed into the sampling well (Fig. 2d).

The process of collecting soil leachates was performed at certain depths of 50 cm and 90 cm, which were afterwards labeled as S1 and S2, respectively (see Table 1). In this study, different irrigation conditions including a sunny hot day, during artificial irrigation, after artificial irrigation, after rainfall with standing surface water, and after rainfall without standing surface water (Table 1). The aforementioned conditions were typically selected due to their notable influence on soil water contents. To ensure the accuracy and reproducibility of the experiment, five replicates were conducted for each sub-categorized soil leachate.

**Table 1:** Sampling conditions and experimental design for leachate collection (each leachate was collected in quintuplicate).

Modes	Sampling conditions	Soil depth (50 cm)	Soil depth (90 cm)
1	Sunny hot day	S1-1	S2-1
2	During artificial irrigation	S1-2	S2-2
3	After artificial irrigation	S1-3	S2-3
4	After rainfall with standing surface water	S1-4	S2-4
5	After rainfall without standing surface water	S1-5	S2-5

By employing a negative air pressure pump, the soil leaching solution can be effectively drained from the porous ceramic probe and then transferred, ultimately, to the temporary bottle from the pipeline (see Fig. 2a). Afterwards, a positive air pressure pump was applied to the temporary bottle, causing the soil leaching solution to flow towards the in-situ monitoring.

Two FDR soil water content sensors (SWCS) were deployed in different soil layers to measure soil water content in order to launch this automatic photochemical flow analysis monitoring system automatically^32^. The porous ceramic probes facilitated the upper and lower pipes from the soil. Subsequently, the upper pipe was linked to the positive air pressure pipe through the pipe T1 and the multi-channel switching valve V1, in which the closed state was predominantly maintained. The pipe T3 was employed to gather the soil leaching solution to the temporary bottle. The pipe T2 was connected to the multi-channel switch valve V2 with a peristaltic pump, which was designed to transfer the leaching solution to the automatic photochemical flow analysis monitoring unit. The pipe T4 and multi-channel switching valve V3 was successfully implemented, wherein the negative air pressure pump creating a pressure of − 90 kPa effectively drain the soil leaching solution from the porous ceramic probe and turn it into the temporary bottle. The ultrasonic liquid position sensor mounted on the bottom of the temporary bottle was responsible for quantifying the volume of the leaching solution that has been accumulated instantaneously. Once the volume of soil leaching solution collected reaches 20 mL, the subsequent the ultrasonic liquid position sensor was initiated by ensuring that the required amount of solution is available. The negative air pressure channel is hermetically sealed, while the solenoid valves V1 and V2 are in an activated state. Furthermore, the positive air pressure channel and peristaltic pump are engaged. The application of positive air pressure facilitated the displacement of any remaining solution within the pipeline, ensuring the complete removal of residue from both the porous ceramic probe and the pipeline. Simultaneously, the peristaltic pump facilitated the transportation of the soil leaching solution from the temporary bottle through pipeline T2 to the automatic photochemical flow analysis monitoring for automated detection of nitrogen and phosphorus levels.

## Design of the automatic photochemical flow analysis system

### The photochemical flow analysis system

**Figure 3:**
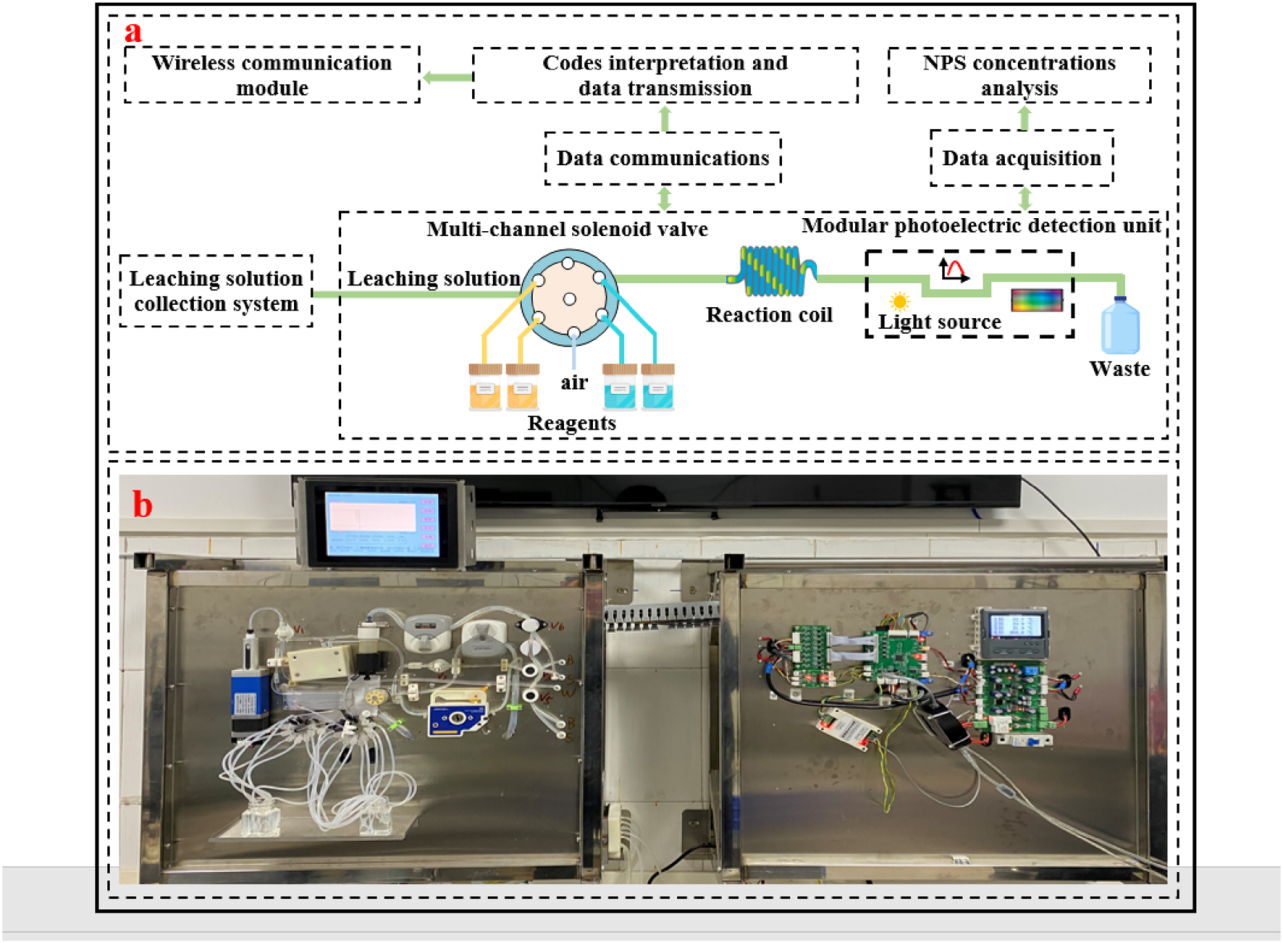
The automatic photochemical flow analysis. (**a**) The schematic diagram of in-situ photochemical flow analysis. (**b**) Prototype of the automatic photochemical flow analysis.

The schematic diagram of automatic photochemical flow analysis is shown in Fig. 3a. The soil leaching solution is transported to the multi-channel solenoid valve by the peristaltic pump, and the multi-channel solenoid valve will circulate the leaching solution and reagent into the reaction coil, and the leaching solution and reagent will completely and thoroughly react in the coil.

The chemical color reaction method employed in automatic photochemical flow analysis for the determination of ammonium nitrogen is known as the indophenol blue process. The reaction between the ammonium ion, hypochlorite, and phenol in a highly alkaline environment results in the formation of a vibrant blue-green dye known as indophenol blue^33^. The primary method employed for the determination of nitrate nitrogen is the phenoldisulfonic acid colorimetric method^34^. This method involves the reaction between the nitrate ion and a phenoldisulfonic acid reagent under anhydrous conditions. Following alkalization, the resulting reaction product forms a stable yellow solution. The predominant method employed for the colorimetric determination of phosphate involves the utilization of the molybdenum blue chemical color reaction. In the presence of an acidic environment, the chemical reaction between phosphoric acid and ammonium molybdate takes place, resulting in the formation of ammonium phosphomolybdate. This compound is characterized by its distinct yellow precipitate. However, it should be noted that under conditions where the phosphorus content is relatively low, the precipitation of ammonium phosphomolybdate does not occur. In order to detect low concentrations of phosphorus, it is imperative to reintroduce a reducing agent to facilitate the reduction of ammonium phosphomolybdate to form blue molybdenum blue. This process is elucidated by chemical Eqs. (1), (2).

1 $${\text{21NH}}_{{4}}^{ + } {\text{ + 12Mo}}_{{7}} {\text{O}}_{{{24}}}^{{6 - }} {\text{ + 7PO}}_{{4}}^{{3 - }} {\text{ + 72H}}^{ + } { = 7}\left( {{\text{NH}}_{{4}} } \right)_{{3}} \left[ {{\text{P}}\left( {{\text{Mo}}_{{3}} {\text{O}}_{{{10}}} } \right)_{{4}} } \right]{\text{ + 36H}}_{{2}} {\text{O}}$$

2 $${\text{8H}}^{ + } { + }\left[ {{\text{P}}\left( {{\text{Mo}}_{{3}} {\text{O}}_{{{10}}} } \right)_{{4}} } \right]^{{3 - }} {\text{ + C}}_{{6}} {\text{H}}_{{8}} {\text{O}}_{{6}} { = }\left[ {{\text{P}}\left( {{\text{Mo}}_{{3}} {\text{O}}_{{9}} } \right)_{{4}} } \right]^{{3 - }} {\text{ + C}}_{{6}} {\text{H}}_{{6}} {\text{O}}_{{6}} {\text{ + 4 H}}_{{2}} {\text{O}}$$

After the complete reaction, different reaction liquids flow into the photoelectric detection unit for absorbance measurement of the reaction solution, and the concentration of the tested sample is determined after data analysis. After testing, the reaction liquid flows down the pipe into the waste liquid tank. After each round of reaction is complete, the multi-channel solenoid valve draws distilled water to clean the inner wall of the pipeline to ensure the accuracy of the next test result (see Fig. 3b).

### Control circuit design and fabrication

**Figure 4:**
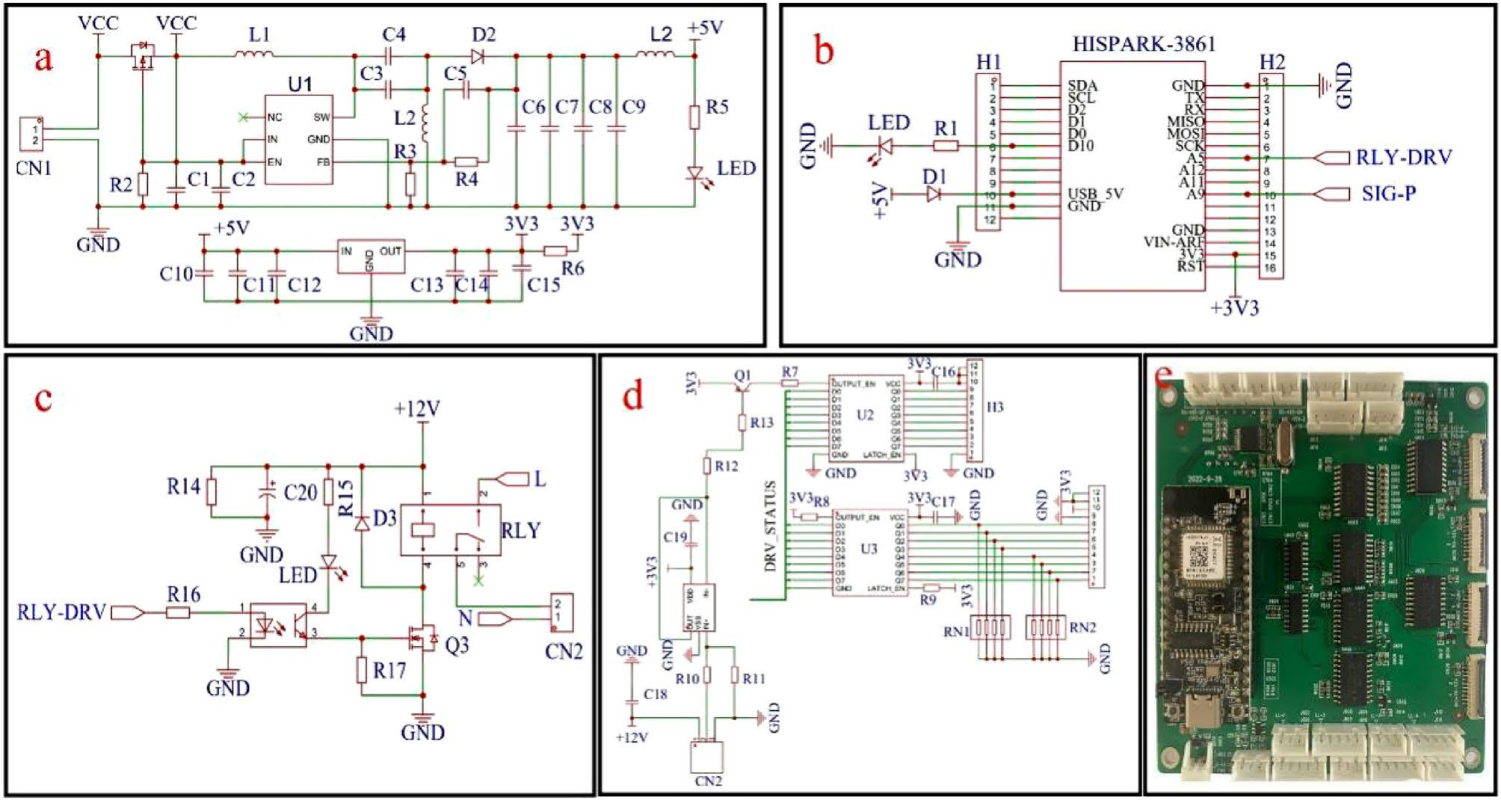
Control circuit design schematic drawings. (**a**) Power supply circuit. (**b**) HISPRK3861 microprocessor. (**c**) Relay driving circuit. (**d**) Pressure monitoring and solenoid valve drive circuit. (**e**) Prototype of the circuit board with HISPRK3861 microprocessor.

The control circuit of this in-situ leachate extraction system shown in Fig. 4 is activated when the volumetric soil water contents is exceeded 19% as measured by a buried FDR soil water content sensor. The leaching solution was delivered out through the porous ceramic probe under a negative air pressure. To sustain the current for the control circuit, a power supply unit module that can provide 3.3–24V voltage was created (see Fig. 4a). The control circuit employed an HISPARK3861 microprocessor chip (refer to Fig. 4b) running on the Harmony OS for acquiring signals from the buried FDR soil water content sensor. The HISPARK3861 microprocessor was specifically engineered to include a total of 15 general purpose input/output (GPIO) pins, with a specific allocation of seven pins dedicated to analog-to-digital (A/D) converter channels. Here, an alternation working mode with a pair of negative and positive air pressure pumps was run by the relay controlled through the HISPARK3861 microprocessor (see Fig. 4c). The soil leaching solution was successfully guided to a temporary storage bottle. The negative air pressure pumps and solenoid valve were controlled using the pressure monitoring circuit (Fig. 4d), which worked intermittently. Specifically, the control circuit activated the relay to launch the negative air pressure pump when the air pressure inside the pipeline exceeded − 70 kPa. This process continued until the pressure dropped below − 90 kPa. The soil leaching solution was then transferred to the temporary storage bottle using the porous ceramic probe and pipelines. Once the negative pressure pump was operated continuously for a duration exceeding 5 min, a protective mode was programmed to activate the negative air pressure pump again in order to keep it safe.

## Results and discussion

Based on a set of five sampling observations, it was ascertained that the shallow soil water content consistently shown lower levels in comparison to the deep soil water content. Furthermore, the inefficacy of the porous ceramic probe in extracting a sufficient quantity of leaching solution for subsequent measurements was observed when the volumetric soil water content (VSWC) dropped below 19%. This phenomenon was observed in cases where the depth of the soil was restricted. Upon reaching the threshold, there was a significant reduction in the duration for subsequent extractions. The decrease in the duration of the extraction process enabled the extraction of significant quantities of soil leaching solution. When the water content of the first and second layers reached 20.07% and 28.05%, the extraction system extracted 105 mL and 165 mL of soil leaching solution, respectively, from the first and second layers within 180 min. For instance, it was noted that when irrigation was implemented on agricultural land and regular rainfall occurred, a consistent amount of 10 mL of leaching solution could be collected during an average time span of 20 min, regardless of the extraction depth. Subsequently, the solution was transferred to the automatic photochemical flow analysis monitoring for the purpose of conducting nitrogen and phosphorus analysis. The soil leaching solution was transported through the pipeline and subsequently introduced into the in-situ monitoring facility for an automated assessment of its nitrogen and phosphorus concentrations. The solution in the automatic photochemical flow analysis monitoring unit was propelled by means of the peristaltic pump. This approach effectively reduced both transportation expenses and the extent of soil leachate degradation.

**Figure 5:**
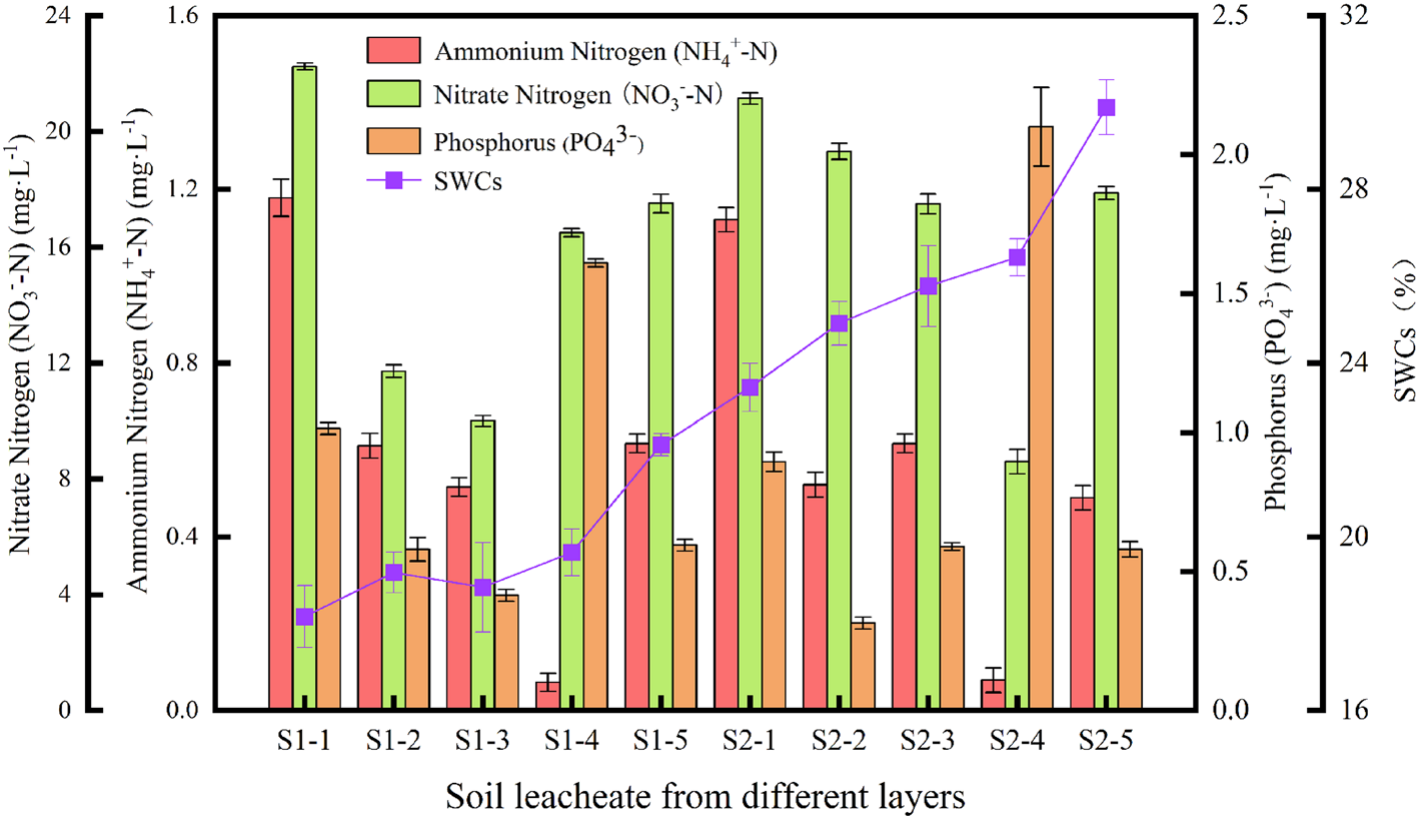
Result plots of N and P measurements with SWCs for different soil leachates.

The findings of the soil leachate extraction design indicated that soil leachates were collected from areas with varying levels of soil water contents, ranging from drier conditions to more significant flooding. The analysis of SWC demonstrates a noticeable upward trend, as depicted in Fig. 5. The R^2^ values associated with the increasing SWCs not only confirm the validity of the experimental design but also provide support for it. This allowed for a more comprehensive investigation into the impact of surface water flooding at depths of 50 cm and 90 cm. Moreover, this observed pattern indicated a significant increase in the moisture levels of the soil at various depths. Given the observed increase in the solubility of nitrogen and phosphorus in water, it is reasonable to hypothesize that the distribution of these elements in the soil may correspond to this phenomenon^35^. Furthermore, the significant increase in soil water content observed in both the upper and deeper soil layers can be attributed to a combination of natural and artificial irrigation.

The primary source of ammonium nitrogen (NH_4_^+^-N) in soil is derived from the microbial decomposition of organic matter within the uppermost layer of soil. The flux of organic material in the uppermost layer of soil consistently exhibits a notable increase in comparison to deeper soil layers^36^. Hence, the results of our analysis comparing the NH_4_^+^-N concentrations revealed that starting from S1-1 (1.15 mg/L) the NH_4_^+^-N run off towards S2 was observed. More specifically, the application of artificial irrigation the soil leaching solution S1-2 and S1-3 were measured to have 0.59 mg/L and 0.53 mg/L NH_4_^+^-N concentrations respectively. The deeper run off to 90 cm was also observed for soil leachates S2-2 to be 0.54 mg/L and S2-3 as 0.60 mg/L. This phenomenon elucidates the rapid depletion of NH_4_^+^-N within the soil profile, specifically between the depths of 50 cm and 90 cm^37^. Following the natural irrigation from S1-4 to S1-5, the NH_4_^+^-N levels experienced an increase due to the presence of dissolved NH_4_^+^-N in the rainwater. In this instance, the precipitation led to the runoff of NH_4_^+^-N from a depth of 50 cm to 90 cm at locations S2-4 and S2-5, similar to the occurrence observed during the artificial irrigation in soil leachates S1-2 and S1-3. Therefore, the observed patterns of both S1 and S2 for NH_4_^+^-N exhibit a strong correlation, providing a comprehensive explanation for the effective drainage and movement of NH_4_^+^-N following both natural and artificial field irrigation events.

The parameter of nitrate nitrogen (NO_3_^−^-N), which is significant in the analysis of soil fertility. Particularly in the case of deeper soil layers, the concentration of nitrate nitrogen (S1-1 22.16 mg/L) in the plot shown in Fig. 5 consistently displays a statistically significant elevation in comparison to NH_4_^+^-N (S1-1 1.15 mg/L). This striking difference can be explained by two main factors: increased runoff of NH_4_^+^-N and biological conversion of NH_4_^+^-N into NO_3_^−^-N through nitrogen fixation in deeper soil layers. Simultaneously, the presence of an alkaline soil environment constitutes an additional influential element that enhances the volatility of NH_4_^+^-N, thereby leading to diminished outcomes. Phosphorus exhibited comparatively reduced mobility in both the upper and deeper layers of soil. An initial runoff phenomenon can be observed following both natural and artificial irrigation practices. However, it has been observed that phosphorus (PO_4_^3−^) exhibits significantly lower mobility compared to ammonium nitrogen (NH_4_^+^-N) and nitrate nitrogen (NO_3_^−^-N). Therefore, the likelihood of PO_4_^3−^ run-off is lower when compared to NH_4_^+^-N and NO_3_^−^-N due to its reduced mobility and stronger interaction with the soil^38^. In relation to irrigation practices, it has been determined that there is a connection between runoff containing nitrogen and phosphorus from the upper and lower layers of that soil. Furthermore, measurements were carried out in a laboratory located within the university in order to compare routine analysis with monitoring that was carried out in the field.

**Figure 6:**
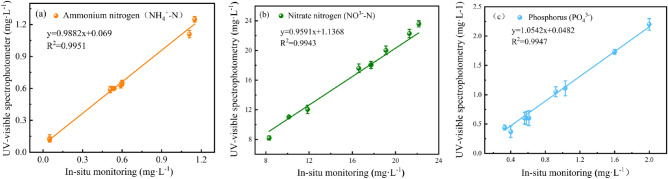
Comparison of measured values by in-situ photochemical flow analysis monitoring system and UV–visible spectrophotometer. (**a**) Ammonium nitrogen (NH_4_^+^-N). (**b**) Nitrate nitrogen (NO_3_^−^-N). (**c**) Phosphorus (PO_4_^3−^).

The conventional UV–visible spectrophotometry was also conducted on the quintuplicate soil leachates that were transported to the laboratory for analysis. The purpose of conducting this activity was to showcase the resilience, accuracy, and effectiveness of the automated measurements conducted within the automatic photochemical flow analysis monitoring. Figure 6 displays the results obtained from an experimental investigation that sought to compare and contrast two distinct methodologies. The data presented in Fig. 6a clearly indicates that there is no significant difference between the quantitative results obtained from laboratory assessment and in-situ monitoring of NH_4_^+^-N. This is evidenced by the absence of a statistically significant disparity between the two datasets. Additionally, the results from automatic photochemical flow analysis monitoring system presented the average concentration ranges of NH_4_^+^-N, NO_3_^−^-N, PO_4_^3−^ are 0.584 mg/L, 15.7 mg/L, 0.844 mg/L, and 0.562 mg/L, 16.828 mg/L and 0.878 mg/L at depths of 50 cm and 90 cm below the soil surface, respectively. Furthermore, the R^2^ values of 0.9951, 0.9943 and 0.9947 obtained, respectively in Fig. 6 for the NH_4_^+^-N, NO_3_^−^-N, and PO_4_^3−^ testing demonstrate a strong correlation between the two quantitative techniques employed. This assertion holds true as it has been demonstrated that all three of these variables exhibit a positive correlation. This correlation provides further substantiation for the objective of this study, which aimed to advance the implementation of automatic photochemical flow analysis monitoring of NH_4_^+^-N, NO_3_^−^-N, and PO_4_^3−^. Furthermore, it has been firmly established, based on the experimental findings obtained from both methodologies, that measurements generated in automatic photochemical flow analysis monitoring system are comparable to those obtained through the UV–visible spectrophotometry. When measured by UV–visible spectrophotometer, the absorption peaks of ammonium nitrogen (NH_4_^+^-N), nitrate nitrogen (NO_3_^−^-N) and phosphorus (PO_4_^3−^). exist at 660 nm, 550 nm and 880 nm, respectively.

## Conclusions

This article has provided a comprehensive overview of a highly effective and innovative flow analysis system designed for the evaluation of non-point source pollutants, specifically NH_4_^+^-N, NO_3_^−^-N, and PO_4_^3−^. The system described in this study demonstrates a robust and in-situ approach to analyzing flow, enabling efficient and accurate identification of these pollutants. By automating the detection process, this system offers a significant advancement in the field of environmental monitoring and pollution control. The experimental results obtained from this study indicate that the average concentrations of NH_4_^+^-N, NO_3_^−^-N, and PO_4_^3−^ were found to be 0.584 mg/L, 15.7 mg/L, and 0.844 mg/L, respectively, at a depth of 50 cm below the soil surface. Similarly, at a depth of 90 cm below the soil surface, the average concentrations of NH_4_^+^-N, NO_3_^−^-N, and PO_4_^3−^ were determined to be 0.562 mg/L, 16.828 mg/L, and 0.878 mg/L, respectively. These findings provide valuable insights into the distribution of these nutrients at different soil depths and contribute to our understanding of nutrient dynamics in the soil environment. In conclusion, the implementation of this innovative methodology has effectively addressed the challenges associated with monitoring non-point source pollution in agricultural settings, particularly in relation to the cumbersome task of measuring and ensuring compliance with protocols for soil leachate transportation.

## Data Availability

The datasets used and/or analyzed during the current study available from the corresponding author on reasonable request.
